# A Literature Review of Vertical Dimension in Prosthodontics Theory and Practice - Part 1: Theoretical Foundations

**DOI:** 10.7759/cureus.61903

**Published:** 2024-06-07

**Authors:** Mostafa I Fayad

**Affiliations:** 1 College of Dentistry, Taibah University, Madinah, SAU; 2 Dentistry, Al-Azhar University, Cairo, EGY

**Keywords:** removable-fixed partial denture, full mouth reconstruction, oral rehabilitation, prosthodontics, vertical dimension

## Abstract

Vertical dimension (VD) is a critical factor in prosthodontics, playing a pivotal role in the functional and aesthetic outcomes of dental treatments. This literature review explores theoretical foundations and the various aspects of VD, including its definition, measurement, and clinical significance in prosthodontics. The relationship between VD and temporomandibular disorders (TMDs) is examined. Additionally, the impact of VD on facial proportions and aesthetics is significant, as it affects the lower third of the face and influences the patient's overall appearance and self-esteem. In conclusion, understanding the intricate relationship between VD, TMDs, facial aesthetics, and psychological well-being is essential for effective prosthodontic treatment. This comprehensive review provides valuable insights into the multifaceted role of VD in enhancing both functional and aesthetic outcomes, ultimately improving patient satisfaction and quality of life.

## Introduction and background

The occlusal vertical dimension in prosthodontics refers to the measurement of the height of the lower face when the upper and lower teeth are in maximum intercuspation [1].

This measurement is crucial in the fabrication of restorations, particularly for edentulous patients. It helps ensure proper alignment and functionality of the prosthesis, as well as esthetic harmony with the patient's natural facial proportions. Without accurate determination of the vertical dimension of occlusion, restorations may result in discomfort, compromised function, and possible degenerative changes in the jaw joint [2]. Therefore, establishing and restoring the vertical dimension is a critical step in prosthodontic treatment to achieve successful rehabilitation outcomes [3].

The vertical dimension can be affected by various factors, such as tooth wear, loss of posterior teeth, skeletal disharmony, and tooth abrasion and attrition. These factors can lead to a decrease in the vertical dimension, which may require restoration or reestablishment in order to restore proper occlusion and function [4-6].

To determine the correct vertical dimension, various techniques and methods have been proposed. These include using pre-existing prostheses, photographs, phonetics, physiological rest position, swallowing, craniometric measurements, radiographic images, and cephalometry. Each technique has its own advantages and limitations, and the choice of method depends on the specific case and the clinician's preference and expertise [1,7,8].

Despite the variety of proposals for inserting a prosthetic at the correct vertical dimension at occlusion (VDO) within the literature, there is no universal agreement on how to achieve this [9]. The scope of this review primarily focuses on the significance of vertical dimension in the field of prosthodontics and the various techniques discussed in the literature to achieve it. The review aims to compile and discuss the available research and expert opinions in the literature regarding the vertical dimension.

A literature search was performed in databases such as PubMed, Cochrane Library, and Google Scholar using the keyword "vertical dimension." This search aimed to identify relevant studies on the vertical dimension of occlusion and its impact on patient quality of life. The search terms included combinations of "vertical dimension" and "occlusal vertical dimension". Articles published in peer-reviewed journals were considered.

The review starts by explaining what vertical dimension is and providing some foundational knowledge about it, which prepares the reader for a deeper understanding as the text progresses to the main body. The significance of the vertical dimension is discussed in a logical and empirical approach, starting with why it is important for prosthodontists to consider the vertical dimension. Part 2 will cover the various methods used to establish the vertical dimension. Part 3 will discuss the loss of the vertical dimension of occlusion and its management implications.

## Review

Definitions

The vertical dimension is defined as the distance measured in a straight line between two selected anatomic points [10]. The vertical dimension at occlusion (VDO) is defined as "the length of the face when the teeth (occlusal rims, central-bearing points, or any other stop) are in contact and the mandible is in centric relation or the teeth are in centric occlusion" [11].

Vertical dimension at rest (VDR) is defined as "the length of the face when the mandible is in rest position". This is the position of the mandible in relation to the maxilla when the muscles are in a state of tonic equilibrium [11]. This position is influenced by the muscles of mastication, which are involved in speech, deglutition, and breathing. The rest position is achieved when there is a balance between gravity and the resting muscle tone. The physiologic rest position is accurately referred to as "range of posture" rather than a single rest position because electromyography (EMG) activity indicates that the clinical rest position does not correspond to minimal muscle activity, which is lower than the clinical rest position by several millimeters [12].

Evolution of the concept of vertical dimension in prosthodontics

The concept of vertical dimension (VD) in prosthodontics has evolved significantly over the decades. This evolution reflects advances in dental materials and techniques, and an improved understanding of the physiology and biomechanics of the masticatory system [13,14].

Historically, the concept of VD was closely associated with the fabrication of complete dentures. Techniques were developed to establish a functional and aesthetically pleasing VD for edentulous patients. Swenson described ten methods for determining VD and centric relation, reflecting the complexity and variability in approaches [15].

Early studies and practices defined VD as the distance between two selected anatomic points, typically the tip of the nose and the chin when the teeth are in occlusion. This simple measurement laid the groundwork for more detailed explorations into its clinical implications [7].

During the mid-20th century, anthropometric methods gained prominence. These methods involved detailed measurements of facial landmarks to determine VD, with studies indicating significant variability among individuals. The use of cephalometric radiographs allowed for more precise assessments of craniofacial structures. This advancement provided a better understanding of the skeletal relationships that underpin VD, although it required specialized equipment and expertise​​ [7,10].

By the late 20th century, the emphasis shifted towards understanding the functional and physiological aspects of VD. Researchers recognized that VD is not a static measure but one influenced by the dynamics of the dentoalveolar complex and masticatory muscles. The concept of dentoalveolar compensation emerged, highlighting that the body adapts to tooth wear by erupting teeth and remodeling alveolar bone to maintain VD. This understanding was crucial for developing more conservative treatment strategies [1,16].

Modern prosthodontics employs a combination of techniques to assess VD. These include facial measurements, cephalometric analysis, phonetic assessments, and clinical judgment. The use of multiple methods ensures a more accurate and individualized determination of VD. Contemporary approaches emphasize the importance of patient adaptation to changes in VD. Gradual increases in VD, using provisional restorations, help patients adjust to new occlusal relationships, reducing the risk of discomfort and dysfunction​​ [17-19].

Anatomical and physiological considerations of vertical dimension

Understanding the anatomical and physiological aspects of vertical dimension is essential for accurate assessment and effective rehabilitation, ensuring optimal function and aesthetics for patients.

Anatomical Considerations

The vertical dimension is influenced by the overall structure and alignment of the craniofacial skeleton, including the maxilla, mandible, teeth, temporomandibular joint, and surrounding musculature. The relationship between these structures determines the vertical dimension of occlusion and influences the stability and comfort of the prosthetic restorations [14,20].

The dentoalveolar complex, including the teeth and alveolar bone, plays a crucial role in maintaining VD. Changes in tooth structure, such as wear or loss, can significantly alter the vertical dimension [21,22].

The third component is the facial musculature. There are eight pairs of muscles that actively maintain the vertical dimension, and these have many different functional activities to perform in addition to this role [23]. In cases in which it is necessary for the vertical dimension to be altered, a period of stabilization in the new dimension is required before a final decision can be made as to the correct occlusal arrangement. This may take several weeks, perhaps even four to six weeks, depending on the patient [24].

Physiological Considerations

The mandibular rest position is the natural, relaxed position of the mandible when the head is upright and the muscles of mastication are in a state of minimal contraction [11].

This position is typically greater than the occlusal vertical dimension (OVD). However, with the absence of teeth, the soft tissues may become hardened, and the muscles may undergo "adaptive shortening," which results in the loss of vertical dimension. The opening and closing muscles tend to be in a state of minimal tonic contraction. This determines the vertical jaw relation. Muscles that produce elevation of the mandible (closing muscles) and gravity also help to control the tonic balance that maintains the physio­logic rest position [25].

Dentoalveolar compensation helps maintain vertical dimension through the eruption of teeth and remodeling of alveolar bone, which helps maintain a stable VD despite wear and other alterations. As the teeth wear down due to bruxism, the vertical dimension is lost, which results in over-closure of the mandible and hence leads to compensatory eruption of teeth and excessive growth of the alveolar bone. In fact, tooth eruption may also compensate for the loss of vertical dimension due to tooth wear, but it is only to a certain extent [26].

The temporomandibular joints' (TMJ) health significantly influences the vertical dimension. TMJ disorders can lead to changes in the vertical dimension. For example, patients with TMDs may adopt compensatory postures or adjust their occlusion to reduce discomfort. This adjustment can lead to increased mechanical wear on tooth surfaces, which may progress to pathological tooth wear (PTW) due to parafunctional mandibular movements, thereby secondarily affecting the vertical dimension [27].

In the field of oral health, numerous researchers have reported an association between malnutrition and impaired growth, affecting the development of facial bones. Malnutrition has been linked to a reduction in the length of the skull base and jaw height. Additionally, variations in maxillomandibular width, lower facial height, and dental and skeletal ages have been observed as consequences of malnutrition [28,29].

Significance of vertical dimension in prosthodontics

Functional Considerations

Proper VD ensures efficient chewing and swallowing. An optimal vertical dimension allows for the correct alignment and contact of the teeth during mastication, facilitating effective grinding and breakdown of food. When the height of occlusion is properly restored, the mandible is allowed to close into the position of retruded contact and the muscles of mastication have more room to work. This maximizes the strength in the masticatory cycle and allows the elevator muscle groups to function properly by providing sufficient intercuspation space for the lower jaw [23].

On the other hand, when the height of occlusion is decreased, a reduction in the masticatory efficiency is observed. It is frequently observed in individuals who suffer from chronic attrition and when the space between the nose and chin is decreased [30].

Additionally, VD plays a crucial role in speech, as it influences the position and movement of the tongue and lips, affecting phonetics and clarity of speech. Researchers found that difficulties pronouncing the sounds "b", "m", "p", "f", and "v" were most commonly related to an increased vertical dimension [31].

TMJ Health

The TMJs rely on a balanced VD to function correctly. An optimal VD helps maintain a stable and comfortable position for the TMJs, reducing the risk of disorders such as TMJ pain, clicking, or dysfunction [24].

The findings on the relationship between vertical dimension (VD) and temporomandibular disorders (TMDs) are varied across different studies.

Some research indicates that both excessive and insufficient VD can strain the TMJs, leading to discomfort and joint issues. The reduced VD is often accompanied by steepening of the gonial angle and can lead to painful symptoms of occlusal disease in the temporomandibular joint. Insufficient stomatognathic function may also present in the form of parafunction, such as nocturnal bruxism, gnashing, and grinding of the teeth. This can cause irreversible damage to teeth and supporting structures. Moreover, chronic myofascial pain of the masticatory and neck muscles may result. [30].

A study by Constantinescu et al. [32] indicates that restoring the lost OVD can improve occlusal relationships and aesthetics without necessarily exacerbating TMDs. The study highlights a case where a 7 mm increase in OVD harmonized the facial profile and functional occlusion, with no adverse TMD symptoms reported.

A systematic review was conducted on several studies reported that a freeway space of 2mm has been suggested as physiologic space. It was therefore indicated that a freeway space of more than 2mm allows for the vertical dimension of occlusion (VDO) to be safely increased. However, these studies also reported that patients could adapt successfully even when the VDO was increased beyond the freeway space (FWS). This suggests that increasing the VDO, traditionally considered risky, can be safe and well-tolerated by patients up to 5mm [8].

Moreno-Hay and Okeson [33] concluded that increasing OVD by less than 5 mm does not lead to the development, aggravation, or perpetuation of TMD symptoms. They found that the stomatognathic system adapts to these changes, although some patients might experience mild, transient symptoms that are typically self-limiting and not of major consequence.

De Melo et al. [34] concluded that the stomatognathic system can adapt to changes in OVD and that a reduction in OVD does not necessarily lead to TMD symptoms. This suggests a degree of flexibility in the system's response to OVD changes.

Aesthetic Considerations

Facial proportions and facial aesthetics are significantly influenced by VD by affecting the lower third of the face. An appropriate VD contributes to a balanced and harmonious facial appearance, impacting features such as lip support, chin projection, and overall facial height. Changes in VD can lead to noticeable alterations in facial aesthetics, impacting a patient's appearance and self-esteem [35].

The correct VD provides support for the facial soft tissues, including the lips and cheeks. Adequate VD helps maintain the natural contour and fullness of the face, preventing a sunken or aged appearance that can result from a reduced vertical dimension [36].

Psychological Impact

The aim of prosthodontic treatment is not only to restore oral function but also for the well-being of the patient's quality of life, happiness, and self-esteem. A patient with vertical dimension loss frequently suffers from depression, lower self-esteem, and more problematic personal and social interactions. If the patient's psychological condition is not recognized and satisfied during the treatment procedure, the outcomes will be more negative [37].

Last but not least, a new clinical study of psychological assessments of patients with vertical dimension loss and their choices in treatment options might be carried out in the future, and this will help to set up the foundation for specific psychological needs and requirements in assisting treatment planning of patients with vertical dimension loss and will energize the development and utilization of brand new and innovative prosthodontic treatment strategies in the near future.

## Conclusions

The vertical dimension is a fundamental aspect of prosthodontics, crucial for both functional and aesthetic outcomes. Proper management of VD is essential for maintaining oral health, ensuring efficient mastication, and achieving harmonious facial aesthetics. Understanding the effects of both increasing and decreasing VD helps clinicians make informed decisions to optimize patient care and improve overall treatment success.
